# MicroRNA Expression Signatures of Bladder Cancer Revealed by Deep Sequencing

**DOI:** 10.1371/journal.pone.0018286

**Published:** 2011-03-28

**Authors:** Yonghua Han, Jiahao Chen, Xiaokun Zhao, Chaozhao Liang, Yong Wang, Liang Sun, Zhimao Jiang, Zhongfu Zhang, Ruilin Yang, Jing Chen, Zesong Li, Aifa Tang, Xianxin Li, Jiongxian Ye, Zhichen Guan, Yaoting Gui, Zhiming Cai

**Affiliations:** 1 Department of Urology, Peking University Shenzhen Hospital, Shenzhen, China; 2 Guangdong Key Laboratory of Male Reproductive Medicine and Genetics, Peking University Shenzhen Hospital, Shenzhen, China; 3 Beijing Genomics Institute at Shenzhen, Shenzhen, China; 4 Department of Urology, Second Xiangya Hospital, Central South University, Changsha, China; 5 Department of Urology, First Affiliated Hospital, Anhui Medical University, Hefei, China; 6 Institute of Urology, Shenzhen PKU-HKUST Medical Center, Shenzhen, China; 7 Shantou University Medical College, Shantou, China; 8 Department of Urology, Second People's Hospital of Shenzhen, Shenzhen, China; Universität Heidelberg, Germany

## Abstract

**Background:**

MicroRNAs (miRNAs) are a class of small noncoding RNAs that regulate gene expression. They are aberrantly expressed in many types of cancers. In this study, we determined the genome-wide miRNA profiles in bladder urothelial carcinoma by deep sequencing.

**Methodology/Principal Findings:**

We detected 656 differentially expressed known human miRNAs and miRNA antisense sequences (miRNA*s) in nine bladder urothelial carcinoma patients by deep sequencing. Many miRNAs and miRNA*s were significantly upregulated or downregulated in bladder urothelial carcinoma compared to matched histologically normal urothelium. *hsa-miR-96* was the most significantly upregulated miRNA and *hsa-miR-490-5p* was the most significantly downregulated one. Upregulated miRNAs were more common than downregulated ones. The *hsa-miR-183*, *hsa-miR-200b∼429*, *hsa-miR-200c∼141* and *hsa-miR-17∼92* clusters were significantly upregulated. The *hsa-miR-143∼145* cluster was significantly downregulated. *hsa-miR-182*, *hsa-miR-183*, *hsa-miR-200a*, *hsa-miR-143* and *hsa-miR-195* were evaluated by Real-Time qPCR in a total of fifty-one bladder urothelial carcinoma patients. They were aberrantly expressed in bladder urothelial carcinoma compared to matched histologically normal urothelium (p<0.001 for each miRNA).

**Conclusions/Significance:**

To date, this is the first study to determine genome-wide miRNA expression patterns in human bladder urothelial carcinoma by deep sequencing. We found that a collection of miRNAs were aberrantly expressed in bladder urothelial carcinoma compared to matched histologically normal urothelium, suggesting that they might play roles as oncogenes or tumor suppressors in the development and/or progression of this cancer. Our data provide novel insights into cancer biology.

## Introduction

Bladder cancer is one of the most prevalent malignancies in the world. About 357,000 bladder cancer cases were newly diagnosed and 145,000 cancer-related deaths were estimated in 2002 [1]. Urothelial carcinoma of the bladder, the most common histopathologic type of bladder cancer, has a variety of genetic and phenotypic characteristics. Many factors, such as chromosomal anomalies, genetic polymorphisms, genetic and epigenetic alterations, contribute to tumorigenesis and progression of urothelial carcinoma of the bladder [2].

MicroRNAs (miRNAs) are endogenous, noncoding RNA molecules of about 22 nucleotides in length that regulate gene expression [3]. They join the RNA-induced silencing complex to regulate their targeted messenger RNA (mRNA) by repressing mRNA translation and/or directing mRNA cleavage [4]. miRNAs play important roles in normal development, cell growth, differentiation, and apoptosis in mammals [5].

More than half miRNA genes are located in cancer-associated genomic regions or in fragile sites [6]. Aberrantly expressed miRNAs have been shown to be associated with many types of cancers. Both losses and gains of miRNA function contribute to cancer development. miRNAs act as oncogenes or tumor suppressors [7]. Most importantly, different cancer types, stages or differentiation states have unique miRNA expression profiles, suggesting that miRNAs can function as novel biomarkers for cancer diagnosis [8], [9].

Several previous researches used miRNA microarrays with limited and varied probes to profile the miRNA expression in bladder cancer and their results did not always indicate consistent results [10]–[13]. To better understand the role of miRNAs in bladder cancer development and progression, comprehensive analysis of the expression and abundance of miRNAs in this cancer is required. With the merit of the high-throughput deep sequencing technology, genome-wide cancer miRNAs can be quantitatively and accurately determined. Here, we present the genome-wide miRNA profiles in nine pairs of snap-frozen bladder urothelial carcinoma and matched histologically normal urothelium by deep sequencing. We found that a collection of miRNAs were aberrantly expressed in bladder urothelial carcinoma compared to matched histologically normal urothelium, several of which were evaluated by Real-Time qPCR in a total of fifty-one bladder urothelial carcinoma patients.

## Results

### Overview of miRNA profiles

Known miRNA expression files between bladder urothelial carcinoma and matched histologically normal urothelium from each patient were compared to find out the differentially expressed miRNAs. The expression of miRNAs in paired samples were shown by calculating log_2_Ratio. The procedures are shown as below: (1) Normalize the expression of miRNAs in two samples (tumor versus normal) to get the expression of transcript per million (TPM). Normalized expression = Actual miRNA count/Total count of clean reads*1000000. (2) Calculate fold-change and p value from the normalized expression. Then Calculate log_2_Ratio. Fold-change = log_2_Ratio (tumor/normal). We determined 656 differentially expressed known human miRNAs and miRNA antisense sequences (miRNA*s) in miRBase14.0 in nine bladder urothelial carcinoma patients (Table S1).

**Figure 1:**
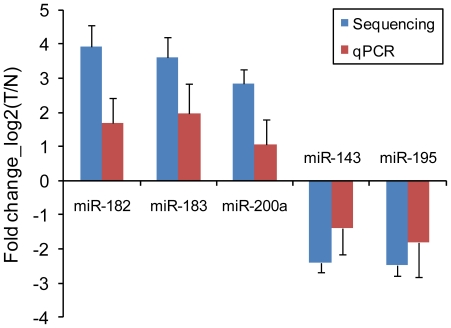
The comparison between deep sequencing data and Real-Time qPCR results. For the comparison between deep sequencing data and Real-Time qPCR results, *hsa-miR-182*, *hsa-miR-183*, *hsa-miR-200a*, *hsa-miR-143* and *hsa-miR-195* determined to be differentially expressed in bladder urothelial carcinoma compared to matched histologically normal urothelium in nine patients by deep sequencing were validated using Real-Time qPCR. The heights of the columns in the chart represent the log-transformed median fold changes (tumor/normal) in expression across the nine patients for each of the five miRNAs validated; the bars represent standard errors. The validation results of the five miRNAs indicated that the deep sequencing data correlated well with the Real-Time qPCR results.

**Figure 2:**
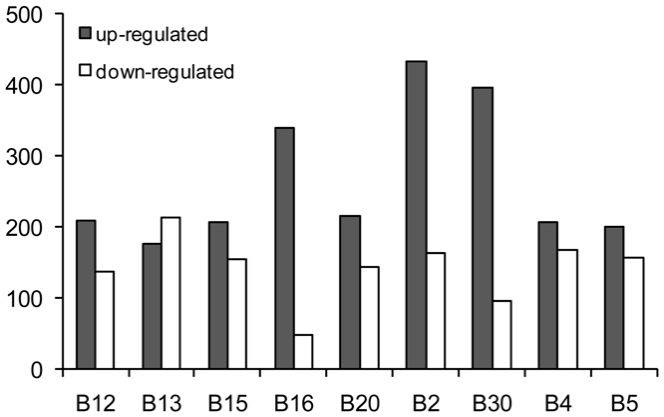
The counts of upregulated and downregulated miRNAs. Many miRNAs were determined to be significantly upregulated or downregulated in bladder urothelial carcinoma compared to matched histologically normal urothelium in nine patients by deep sequencing. The counts of upregulated and downregulated miRNAs varied across the nine patients. In eight out of nine bladder urothelial carcinoma patients upregulated miRNAs were more common than downregulated ones. In only one patient (Patient No. B13), upregulated miRNAs were less common than downregulated ones.

**Figure 3:**
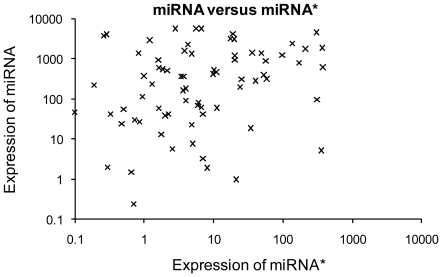
The expression of miRNA and paired miRNA*. A collection of miRNAs and paired miRNA antisense sequences (miRNA*s) were determined to be expressed in nine bladder urothelial carcinoma patients by deep sequencing. We identified a remarkable divergence of expression between miRNA and paired miRNA*. The expression of miRNA/miRNA* pairs is shown in scatter plot with logarithm coordinate system; each point represents the expression of a miRNA/miRNA* pair. In most miRNA/miRNA* pairs, the expression level of miRNA was higher than that of paired miRNA*. In a small number of miRNA/miRNA* pairs, miRNA was less abundant than paired miRNA*.

We identified a great number of miRNAs and miRNA*s that were signicantly upregulated or downregulated in these patients and could discriminate bladder urothelial carcinoma from matched normal urothelium. *hsa-miR-96* (log_2_Ratio = 4.664328) was the most significantly upregulated miRNA and *hsa-miR-490-5p* (log_2_Ratio = −5.79794) was the most significantly downregulated one (Table 1). Selected differentially expressed miRNAs were validated by Real-Time qPCR. The Real-Time qPCR findings correlated well with the sequencing analysis. The comparison between Real-Time qPCR findings and deep sequencing results is shown in Figure 1. The counts of upregulated and downregulated miRNAs varied in different patients. Upregulated miRNAs were more common than downregulated ones (Figure 2). Additionally, we identified a remarkable divergence of expression levels between miRNA and paired miRNA*. The expression levels of miRNAs were usually higher than that of paired miRNA*s (Figure 3).

**Table 1:** A collection of deregulated miRNAs detected by deep sequencing in nine bladder carcinoma patients.

Upregulated in cancer		Downregulated in cancer	
miRNA	log_2_Ratio^a^	miRNA	log_2_Ratio^b^
hsa-miR-96	4.664327577	hsa-miR-490-5p	−5.79794
hsa-miR-182	4.095720336	hsa-miR-99a*	−5.11761
hsa-miR-183	3.820320401	hsa-miR-490-3p	−4.93958
hsa-miR-429	3.071280539	hsa-miR-125b-2*	−4.68986
hsa-miR-141	3.040138264	hsa-miR-99a	−4.00745
hsa-miR-200c	2.780906641	hsa-miR-133a	−4.20124
hsa-miR-200a	2.77846432	hsa-miR-1	−3.97239
hsa-miR-200b	2.747966283	hsa-miR-125b	−3.32189
hsa-miR-18a	2.527819159	hsa-miR-145	−3.15196
hsa-miR-7	2.553138	hsa-miR-195	−2.11536
hsa-miR-25*	2.398581	hsa-miR-143*	−2.93729
hsa-miR-19b	2.075886368	hsa-miR-145*	−2.78189
hsa-miR-19a	2.05391435	hsa-let-7c	−2.51985
hsa-miR-17	1.732520894	hsa-miR-100	−2.37375
hsa-miR-20a	1.644486659	hsa-miR-143	−2.53496

a,b Bladder urothelial carcinoma versus matched histologically normal urothelium, False discovery rate (FDR)≤0.1%, p<0.01.

Besides the known miRNAs and miRNA*s, 92 novel miRNA sequence candidates were detected in our study (Table S2). Most of them were expressed at very low levels and only in certain samples. Their expression patterns and possible roles need further investigation.

### Expression of clustered miRNAs

We found that a collection of deregulated miRNA clusters were expressed. The *hsa-miR-183*, *hsa-miR-200b∼429*, *hsa-miR-200c∼141* and *hsa-miR-17∼92* clusters were significantly upregulated. The *hsa-miR-143∼145* cluster was significantly downregulated.

### Real-Time qPCR validation

*hsa-miR-182*, *hsa-miR-183*, *hsa-miR-200a*, *hsa-miR-143* and *hsa-miR-195* were evaluated by Real-Time qPCR in a total of fifty-one bladder urothelial carcinoma patients. *hsa-miR-182*, *hsa-miR-183* and *hsa-miR-200a* were overexpressed *hsa-miR-143* and *hsa-miR-195* were underexpressed in bladder urothelial carcinoma compared to matched histologically normal urothelium (p<0.001 for each miRNA) (Table S3).

## Discussion

The development of high throughput deep sequencing technology provides the possibility of a near complete view of miRNA profiles. Deep sequencing technology has the potential to identify novel tissue specific miRNAs [14]. It determines the absolute abundance of miRNAs and can discover novel miRNAs that have been missed by common cloning and sequencing methods [15]. Deep sequencing technology is superior to microarrays which determine limited known miRNAs and usually do not contain the full list of known miRNA antisense sequences. Up to now deep sequencing technology has been the gold standard for the comprehensive analysis of miRNAs.

Researches have revealed that miRNAs can be used as biomarkers for different types of cancers [8], [9]. Some miRNAs as biomarkers are able to trace the tissue of origin of cancers of unknown primary origin [16]. Specific miRNA signatures are superior to mRNA signatures in predicting the prognosis of lung cancer [17].

In this work, we used deep sequencing technology to determine the comprehensive miRNA expression profiles in nine pairs of snap-frozen bladder urothelial carcinoma and matched histologically normal urothelium. Many miRNAs were significantly upregulated or downregulated in bladder urothelial carcinoma compared to matched histologically normal urothelium in these patients, suggesting that these aberrantly expressed miRNAs might play roles in these cancers. The most significantly upregulated miRNA *hsa-miR-96* has been reported to be oncogenic [18], but the role of the most significantly downregulated miRNA *hsa-miR-490-5p* is not clear. A lot of miRNA*s were significantly deregulated in bladder urothelial carcinoma compared to matched histologically normal urothelium in this study. Some miRNA*s can join the RNA-induced silencing complex and have inhibitory function [19].

Many deregulated miRNA clusters were expressed according to our deep sequencing analysis. We found that the *hsa-miR-183* cluster was overexpressed in bladder cancer. This cluster consists of *hsa-miR-96*, *hsa-miR-182* and *hsa-miR-183* and is located on chromosome 7. These three miRNAs are upregulated in prostate carcinoma [18]. The coexpression pattern of the miRNAs of this cluster in cancers suggests they might play roles together. The *hsa-miR-200* family of miRNAs (*hsa-miR-200a/b/c*, *hsa-miR-141* and *hsa-miR-429*) were overexpressed in bladder cancer. The *hsa-miR-200b∼429* cluster is located on chromosome 1 and the *hsa-miR-200c∼141* cluster is located on chromosome 12. The coexpression of these clusters suggests that they might be controlled by common factors and play roles together. *hsa-miR-200b*, *hsa-miR-200a* and *hsa-miR-429* miRNAs are encoded by a single polycistronic transcript and negatively regulated by ZEB1 and SIP1 [20]. TGFß 1 can downregulate the *hsa-miR-200* family leading to the upregulation of ZEB1 and ZEB2 [21]. The *hsa-miR-200* family are also overexpressed in ovarian and cervical cancers [22]–[24], suggesting this miRNA family are oncogenic in seveval cancers. The *hsa-miR-17-92* cluster is located on chromosome 13 and acts as oncogenes. E2F1 and E2F3 can directly activate the transcription of these miRNAs [25]. The *hsa-miR-143∼145* cluster is located on chromosome 5 and downregulated in many cancers, including bladder cancers and their cell lines [13], [26]. Our findings provided more evidence to support that the *hsa-miR-143∼145* cluster is tumor-suppressive in bladder cancer.

In this study, Real-Time qPCR was performed to evaluate the expression patterns of *hsa-miR-182*, *hsa-miR-183*, *hsa-miR-200a*, *hsa-miR-143* and *hsa-miR-195* in a total of fifty-one bladder urothelial carcinoma patients. *hsa-miR-182*, *hsa-miR-183* and *hsa-miR-200a* were overexpressed *hsa-miR-143* and *hsa-miR-195* were underexpressed in bladder urothelial carcinoma compared to matched histologically normal urothelium. These findings supported our deep sequencing analysis.

We compared our results to published data to seacrch for independent external validations. *hsa-miR-182*, *hsa-miR-183* and *hsa-miR-224* are upregulated and *hsa-miR-1*, *hsa-miR-101*, *hsa-miR-143*, *hsa-miR-145*, *hsa-miR-127* and *hsa-miR-29c* are downregulated in bladder urothelial carcinoma compared to matched histologically normal urothelium [27]. Our deep sequencing results were largely consistent with these findings. The upregulation of *hsa-miR-182* and *hsa-miR-183* and the downregulation of *hsa-miR-143* were found in bladder urothelial carcinoma compared to matched histologically normal urothelium in our Real-Time qPCR evaluation. The profiling of miRNAs in 106 bladder cancers and 11 normal samples using microarrays has revealed that a set of miRNAs are upregulated or downregulated in bladder cancers [13]. Of these deregulated miRNAs, *hsa-miR-21*, *hsa-miR-20a*, *hsa-miR-184*, *hsa-miR-26a*, *hsa-miR-125b*, *hsa-miR-29a*, *hsa-miR-29c* and so on share the similar expression patterns with our results. *hsa-miR-133a*, *hsa-miR-133b* and *hsa-miR-195* are downregulated in bladder cancers [28]. These miRNAs showed downregulation in our sequencing analysis and *hsa-miR-195* downregulation was comfirmed by Real-Time qPCR in this study.

We used matched adjacent histologically normal urothelium as control in our study. It has been reported that select samples of histologically normal urothelium from bladder cancer patients have genetic alterations [29]. Some chromosomal aberrations shared by both a tumor and a histologically normal looking tissue adjacent to the tumor can cause similar changes of miRNA expression. To compensate for this weakness, we compared our results to several published reports using urothelium from normals as control [10], [13], [28]. A large collection of findings were comparable between theirs and ours. Our findings were also consistent with those papers using matched adjacent histologically normal urothelium as control concerning a lot of miRNAs [26], [27]. Overlapping findings between published data and our results are shown in Table 2.

**Table 2:** Overlapping findings between published data and current results.

Control	miRNA upregulated in cancer	miRNA downregulated in cancer	Reference
Urothelium from normals	hsa-miR-193a-3p, hsa-miR-21,	hsa-miR-143, hsa-miR-145,	[13]
	hsa-miR-20a, hsa-miR-184,	hsa-miR-126*,hsa-miR-26a,	
	hsa-miR-492	hsa-miR-125b, hsa-miR-29a	
Urothelium from normals	hsa-miR-223, hsa-miR-26b,		[10]
	hsa-miR-185, hsa-miR-203,		
	hsa-miR-23a, hsa-miR-205		
Urothelium from normals		hsa-miR-133a, hsa-miR-133b,	[28]
		hsa-miR-195, hsa-miR-145,	
		hsa-miR-125b	
Matched urothelium^a^	hsa-miR-182, hsa-miR-183,	hsa-miR-1, hsa-miR-101,	[27]
	hsa-miR-224, hsa-miR-196a,	hsa-miR-143, hsa-miR-145,	
	hsa-miR-10a, hsa-miR-203	hsa-miR-127, hsa-miR-29c	
Matched urothelium^b^		hsa-miR-143	[26]

a,b Matched histologically normal urothelium from bladder urothelial carcinoma patients.

To the best of our knowledge, this is the first genome-wide profiling of miRNAs in human bladder cancer by deep sequencing. We found that a collection of deregulated miRNAs were aberrantly expressed in bladder urothelial carcinoma compared to matched histologically normal urothelium, suggesting that they might play roles as oncogenes or tumor suppressors in the development and/or progression of this cancer. Our data provide novel insights into cancer biology. More work will be needed to determine the potential roles of miRNAs as diagnostic biomarkers and candidate therapeutic targets for bladder cancer.

## Materials and Methods

### Patient samples

Written informed consent was obtained from all patients and the study was approved by the Institutional Review Board of Peking University Shenzhen Hospital. Fifty-one patients with bladder urothelial carcinoma who received partial or radical cystectomy were included in the study. Of these patients, nine were used for initial deep sequencing analysis of miRNAs and forty-two were used for an extra evaluation. Bladder urothelial carcinoma was diagnosed histopathologically. Bladder urothelial carcinoma and matched histologically normal urothelium from each subject were snap-frozen in liquid nitrogen immmediately after resection. Detailed information of nine bladder urothelial carcinoma patients in deep sequencing set is summarized in Table S4.

### RNA Extraction

When the proportion of cancer cells in a tissue section was greater than 80%, the frozen block was subjected to RNA extraction. Total RNA was extracted from fifty-one pairs of snap-frozen bladder urothelial carcinoma and matched histologically normal urothelium using TRIzol reagent (Invitrogen, Carlsbad, CA, USA) according to the manufacturer's protocol. The RNA integrity was evaluated by Agilent 2100 BioAnalyzer (Agilent Technologies, Palo Alto, CA, USA).

### miRNA sequencing and analysis

Eighteen small RNA libraries prepared from nine pairs of snap-frozen bladder urothelial carcinoma and matched histologically normal urothelium were constructed, amplified and sequenced. Total RNA was used for miRNA sequencing. After 5′adapter and 3′adapter were ligated to small RNAs, Reverse transcription was performed. Then PCR was performed and PCR products were purified. Lastly, miRNA libraries were constructed and sequenced by the Illumina Cluster Station and Genome Analyze (Illumina Inc, CA, USA) at Beijing Genomics Institute at Shenzhen according to the manufacturer's protocol.

Low quality reads were removed and adapter sequences were accurately clipped with the aid of a dynamic programming algorithm before further analysis. After elimination of duplicate reads, the remaining reads of at least 18 nt were mapped to a human reference genome (hg19) using SOAP V2.0 [30]. To identify sequence tags originating from coding exons, repeats, rRNA, tRNA, snRNA, and snoRNA, UCSC RefGene, RepeatMasker, NCBI Refseq data and the ncRNA annotations compiled from the NCBI Genbank data (http://www.ncbi.nih.gov) were used. To identify novel miRNA genes, all hairpin-like RNA structures encompassing small RNA tags were identified using MIREAP (http://sourceforge.net/projects/mireap).

### Real-Time qPCR confirmation and statistical methods

Three overexpressed (*hsa-miR-182*, *hsa-miR-183* and *hsa-miR-200a*) and two underexpressed miRNAs (*hsa-miR-143* and *hsa-miR-195*) were evaluated in all of the patients included in this study. These miRNAs were selected because they were significantly deregulated in the initial deep sequecing analysis. snRNA U6 was used as the endogenous control. Real-Time qPCR was performed using the All-in-One™ miRNA qRT-PCR Detection Kit (GeneCopoiea Inc, Rockville, MD, USA). 10 µg of total RNA was converted to cDNA according to the manufacturer's protocol. PCR was performed in a total reaction volume of 20 µl, including 10 µl of 2xAll-in-One™ qPCR Mix, 2 µl of Universal Adaptor PCR Primer (2 µM), 2 µl of All-in-One™ qPCR Primer (2 µM), 2 µl of First-Strand cDNA (diluted in 1∶5), 50xROX Reference Dye 0.4 µl and 3.6 µl double-distilled water. The reactions were performed and analyzed using the ABI PRISM 7000 Fluorescent Quantitative PCR System (Applied Biosystems, Foster City, CA,USA). PCR reactions were performed for cancer and normal cDNA in triplicate for each set. The cycling parameters for PCR were as follows: (1) an initial denaturation step of 15 min at 95°C; (2) 40 cycles, with 1 cycle consisting of 15 s at 95°C, 20 s at 55°C, and 30 s at 70°C. The catalog numbers of All-in-One™ miRNA qPCR Primers are listed in Table S5. The median in each triplicate was used to calculate relative miRNA concentrations (ΔCt = Ct_medianmiRNA_−Ct_mediansnRNAU6_). Expression fold changes were calculated using 2^−ΔΔCt^ methods [31]. The miRNA expression differences between cancer and control were analysed using Student's *t* test within SPSS (Version 16.0 SPSS Inc.). A value of p<0.05 was considered as statistically significant.

## Supporting Information

Table S1 **miRNAs were differentially expressed between bladder urothelial carcinoma and matched histologically normal urothelium.**
(XLS)

Table S2 **The sequences of novel miRNA candidates were detected in bladder urothelial carcinoma and matched histologically normal urothelium.**
(XLS)

Table S3 **Delt-Ct values of Real-Time qPCR in fifty-one bladder urothelial carcinoma patients.**
(DOC)

Table S4 **Patient information in deep sequencing set.**
(DOC)

Table S5 **Primer catalog.**
(DOC)

## References

[1] Parkin DM, Bray F, Ferlay J, Pisani P (2005). Global Cancer Statistics, 2002.. CA Cancer J Clin.

[2] Kim WJ, Bae SC (2008). Molecular biomarkers in urothelial bladder cancer.. Cancer Sci.

[3] Lagos-Quintana M, Rauhut R, Lendeckel W, Tuschl T (2001). Identificationof novel genes coding for smallexpressed RNAs.. Science.

[4] Bartel DP (2004). MicroRNAs: genomics, biogenesis, mechanism, and function.. Cell.

[5] Alvarez-Garcia I, Miska E (2005). MicroRNA functions in animal development and human disease.. Development.

[6] Calin GA, Sevignani C, Dumitru CD, Hyslop T, Noch E, Y (2004). Human microRNA genes are frequently located at fragile sites and genomic regions involved in cancers.. Proc Natl Acad Sci USA.

[7] Carlo M, Croce (2009). Causes and consequences of microRNA dysregulation in cancer.. Nature Reviews Genetics.

[8] Stenvang J, Silahtaroglu AN, Lindow M, Elmen J, Kauppinen S (2008). The utility of LNA in microRNA-based cancer diagnostics and therapeutics.. Semin Cancer Biol.

[9] Lu J, Getz G, Miska EA, Alvarez-Saavedra E, Lamb J (2005). MicroRNA expression profiles classify human cancers.. Nature.

[10] Gottardo F, Liu CG, Ferracin M, Calin GA, Fassan M (2007). MicroRNA profiling in kidney and bladder cancers.. Urol Oncol.

[11] Neely LA, Rieger-Christ KM, Neto BS, Eroshkin A, Garver J (2010). A microRNA expression ratio defining the invasive phenotype in bladder tumors.. Urol Oncol.

[12] Wang G, Zhang H, He H, Tong W, Wang B (2010). Up-regulation of microRNA in bladder tumor tissue is not common.. Int Urol Nephrol.

[13] Dyrskjøt L, Ostenfeld MS, Bramsen JB, Silahtaroglu AN, Lamy P (2009). Genomic profiling of microRNAs in bladder cancer: miR-129 is associated with poor outcome and promotes cell death in vitro.. Cancer Res.

[14] Rathjen T, Pais H, Sweetman D, Moulton V, Munsterberg A (2009). High throughput sequencing of microRNAs in chicken somites.. FEBS Lett.

[15] Creighton CJ, Reid JG, Gunaratne PH (2009). Expression profiling of microRNAs by deep sequencing.. Brief Bioinform.

[16] Rosenfeld N, Aharonov R, Meiri E, Rosenwald S, Spector Y (2008). MicroRNAs accurately identify cancer tissue origin.. Nat Biotechnol.

[17] Raponi M, Dossey L, Jatkoe T, Wu X, Chen G (2009). MicroRNA classifiers for predicting prognosis of squamous cell lung cancer.. Cancer Res.

[18] Schaefer A, Jung M, Mollenkopf HJ, Wagner I, Stephan C (2010). Diagnostic and prognostic implications of microRNA profiling in prostate carcinoma.. Int J Cancer.

[19] Okamura K, Phillips MD, Tyler DM, Duan H, Chou YT (2008). The regulatory activity of microRNA* species has substantial influence on microRNA and 3′ UTR evolution.. Nat Struct Mol Biol.

[20] Bracken CP, Gregory PA, Kolesnikoff N, Bert AG, Wang J (2008). A double-negative feedback loop between ZEB1-SIP1 and the microRNA-200 family regulates epithelial-mesenchymal transition.. Cancer Res.

[21] Katoh Y, Kato M (2008). Hedgehog signaling, epithelial-to-mesenchymal transition and miRNA (Review).. Int J Mol Med.

[22] Wyman SK, Parkin RK, Mitchell PS, Fritz BR, O'Briant K (2009). Repertoire of microRNAs in epithelial ovarian cancer as determined by next generation sequencing of small RNA cDNA libraries.. PLoS One.

[23] Lee JW, Choi CH, Choi JJ, Park YA, Kim SJ (2008). Altered MicroRNA expression in cervical carcinomas.. Clin Cancer Res.

[24] Witten D, Tibshirani R, Gu SG, Fire A, Lui WO (2010). Ultra-high throughput sequencing-based small RNA discovery and discrete statistical biomarker analysis in a collection of cervical tumours and matched controls.. BMC Biology.

[25] Mendell JT (2008). miRiad roles for the miR-17-92 cluster in development and disease.. Cell.

[26] Lin T, Dong W, Huang J, Pan Q, Fan X (2009). MicroRNA-143 as a tumor suppressor for bladder cancer.. J Urol.

[27] Friedman MJ, liang G, Liu C, Wolff ME, Tsai CY (2009). The putative tumor suppressor microRNA-101 modulates the cancer epigenome by repressing the polycomb group protein EZH2.. Cancer Res.

[28] Ichimi T, Enokida H, Okuno Y, Kunimoto R, Chiyomaru T (2009). Identification of novel microRNA targets based on microRNA signatures in bladder cancer.. Int J Cancer.

[29] Hartmann A, Moser K, Kriegmair M, Hofstetter A, Hofstaedter F (1999). Frequent genetic alterations in simple urothelial hyperplasias of the bladder in patients with papillary urothelial carcinoma.. Am J Pathol.

[30] Li R, Yu C, Li Y, Lam TW, Yiu SM (2009). SOAP2: an improved ultrafast tool for short read alignment.. Bioinformatics.

[31] Schmittgen TD, Lee EJ, Jiang J, Sarkar A, Yang L (2008). Real-time PCR quantification of precursor and mature microRNA.. Methods.

