# Supplementary material for: MicroRNA Expression Signatures of Bladder Cancer Revealed by Deep Sequencing
Source: PLoS One. 2011 Mar 28;6(3):e18286. doi: 10.1371/journal.pone.0018286 (PMC3065473; doi:10.1371/journal.pone.0018286)
Supplement: Table S3 — Delt-Ct values of Real-Time qPCR in fifty-one bladder urothelial carcinoma patients. (DOC) [file pone.0018286.s003.doc]

**Table S3 Delt-Ct values of Real-Time qPCR in fifty-one bladder urothelial carcinoma patients**

| Case No. | hsa-miR-182 | | hsa-miR-183 | | hsa-miR-200a | | hsa-miR-143 | | hsa-miR-195 | |
| --- | --- | --- | --- | --- | --- | --- | --- | --- | --- | --- |
|  | ΔCt-tumor | ΔCt-control | ΔCt-tumor | ΔCt-control | ΔCt-tumor | ΔCt-control | ΔCt-tumor | ΔCt-control | ΔCt-tumor | ΔCt-control |
| B2 | 3.63 | 7.21 | 3.04 | 5.53 | 1.92 | 5.39 | 5.25 | -1.44 | 6.20 | -3.70 |
| B4 | 4.92 | 5.37 | 4.64 | 4.99 | 3.67 | 3.83 | 3.10 | 0.06 | 2.55 | -1.36 |
| B5 | 7.80 | 10.07 | 7.65 | 10.07 | 6.76 | 8.72 | 6.73 | 5.36 | 7.07 | 4.56 |
| B12 | 2.21 | 4.93 | 4.93 | 6.94 | 3.11 | 4.16 | 4.08 | 1.56 | 5.45 | 3.64 |
| B13 | 1.25 | 8.97 | 1.36 | 9.88 | 2.48 | 9.24 | 3.23 | 3.11 | 6.73 | 6.65 |
| B15 | 9.14 | 10.53 | 8.92 | 10.90 | 8.38 | 9.51 | 6.04 | 5.32 | 7.79 | 7.08 |
| B16 | 3.88 | 4.72 | 3.93 | 4.70 | 2.78 | 3.16 | 1.29 | -3.38 | 0.63 | -1.42 |
| B20 | 8.31 | 8.65 | 7.73 | 7.22 | 7.88 | 8.39 | 4.95 | 4.41 | 7.14 | 6.08 |
| B30 | 5.16 | 6.83 | 5.79 | 6.72 | 6.19 | 6.09 | 1.76 | 2.53 | 3.74 | 2.64 |
| B32 | 14.53 | 15.53 | 5.08 | 12.93 | 7.95 | 13.54 | 6.52 | 7.24 | 11.00 | 12.08 |
| B34 | 11.93 | 13.83 | 10.45 | 11.66 | 9.18 | 9.40 | 6.38 | 4.41 | 6.94 | 6.55 |
| B35 | 7.92 | 12.34 | 10.36 | 9.27 | 5.92 | 10.16 | 7.58 | 8.42 | 8.38 | 7.08 |
| B37 | 11.35 | 12.35 | 8.84 | 10.23 | 6.32 | 7.30 | 8.83 | 3.45 | 5.67 | 5.97 |
| B38 | 0.11 | 0.62 | 0.45 | 0.66 | 0.95 | 1.90 | 4.33 | 2.40 | 5.42 | 5.12 |
| B40 | 10.33 | 10.19 | 8.39 | 9.41 | 6.53 | 6.35 | 5.10 | 4.61 | 6.20 | 5.33 |
| B43 | 10.60 | 12.66 | 7.83 | 8.59 | 5.90 | 7.28 | 9.48 | 6.33 | 7.19 | 7.98 |
| B44 | 12.18 | 14.36 | 8.99 | 11.84 | 7.77 | 9.90 | 10.69 | 6.81 | 9.85 | 8.84 |
| B51 | 0.49 | 4.23 | 0.62 | 3.05 | 3.92 | 7.15 | 5.53 | 1.14 | 8.11 | 4.42 |
| B52 | 5.70 | 6.59 | 5.26 | 5.66 | 1.92 | 3.84 | 4.42 | 5.46 | 3.43 | 3.31 |
| B53 | 4.96 | 5.92 | 5.59 | 5.21 | 3.98 | 3.94 | 2.05 | -5.26 | 3.86 | -0.36 |
| B54 | 1.96 | 2.23 | 2.46 | 1.82 | 2.55 | 3.31 | 4.94 | 3.19 | 2.17 | 1.93 |
| B55 | 4.72 | 6.61 | 4.01 | 5.84 | 3.89 | 3.79 | 3.74 | 1.99 | 3.29 | 0.19 |
| B56 | 2.74 | 8.65 | 3.42 | 6.54 | 0.82 | 1.56 | 4.10 | 3.70 | 0.71 | 0.77 |
| B57 | 7.05 | 8.30 | 5.63 | 7.54 | 2.49 | 3.24 | 0.70 | 0.59 | 1.39 | 1.59 |
| B58 | 9.67 | 10.46 | 9.76 | 9.84 | 11.21 | 12.0 | 8.89 | 11.76 | 11.32 | 11.53 |
| B59 | 4.05 | 6.23 | 3.26 | 5.02 | 7.22 | 4.41 | 9.03 | 1.20 | 8.67 | 3.96 |
| B60 | 4.85 | 8.01 | 5.13 | 10.45 | 5.70 | 4.34 | 9.64 | -0.08 | 8.71 | 3.66 |
| B61 | 5.23 | 11.39 | 5.43 | 9.69 | 1.20 | 7.11 | 5.17 | 5.76 | 1.61 | 2.00 |
| B62 | 1.09 | 0.30 | -0.01 | -0.14 | 4.27 | 5.56 | 4.42 | 3.23 | 3.91 | 2.69 |
| B63 | 5.35 | 9.43 | 6.54 | 6.42 | 7.43 | 11.97 | 5.01 | 5.98 | 4.33 | 4.47 |
| B64 | 3.91 | 2.94 | 3.40 | 2.90 | 2.38 | 1.86 | 6.51 | 1.06 | 8.17 | 0.77 |
| B65 | 8.09 | 12.59 | 5.97 | 9.06 | 5.28 | 9.74 | 6.98 | 7.21 | 8.95 | 9.25 |
| B66 | 10.99 | 12.65 | 8.28 | 9.29 | 5.43 | 7.09 | 7.70 | 3.38 | 6.61 | 6.38 |
| B69 | 10.51 | 12.45 | 7.72 | 12.17 | 6.98 | 10.45 | 9.54 | 7.77 | 9.60 | 9.96 |
| B78 | 8.96 | 8.83 | 7.45 | 9.49 | 6.30 | 7.47 | 6.37 | 5.24 | 5.88 | 5.94 |
| B79 | 2.82 | 2.19 | 5.81 | 5.47 | 3.12 | 4.71 | 6.22 | 0.57 | 3.43 | 3.91 |
| B80 | 10.00 | 14.15 | 7.67 | 11.72 | 6.16 | 10.75 | 7.99 | 3.33 | 7.57 | 7.90 |
| B81 | 0.70 | 0.88 | 3.42 | 2.31 | 3.24 | 1.38 | 2.73 | 0.18 | 8.01 | 3.62 |
| B82 | 9.46 | 8.44 | 9.67 | 8.59 | 7.83 | 6.56 | 6.73 | 1.25 | 7.26 | 4.75 |
| B83 | 9.43 | 9.17 | 7.48 | 6.44 | 6.07 | 4.84 | 9.25 | 6.41 | 6.71 | 6.45 |
| B87 | 7.90 | 10.35 | 4.40 | 9.81 | 2.16 | 9.24 | 5.47 | 2.05 | 4.62 | 5.10 |
| B88 | 3.74 | 8.95 | 2.82 | 7.75 | 2.62 | 8.15 | 2.55 | 2.08 | 1.70 | 2.79 |
| B89 | 8.90 | 11.32 | 8.69 | 8.22 | 9.11 | 10.9 | 4.42 | 5.32 | 8.25 | 7.10 |
| B98 | 6.73 | 10.32 | 7.63 | 10.11 | 3.80 | 6.24 | 6.32 | 2.91 | 11.67 | 11.79 |
| B104 | 9.03 | 11.97 | 6.78 | 9.99 | 4.88 | 6.52 | 7.82 | 5.21 | 6.37 | 6.54 |
| B105 | 0.49 | 4.23 | 3.86 | 10.40 | 4.70 | 9.30 | 4.78 | 5.52 | 9.53 | 7.31 |
| B117 | 5.70 | 6.59 | 8.65 | 9.68 | 5.39 | 10.75 | 8.09 | 3.65 | 9.88 | 10.12 |
| B118 | 11.71 | 13.80 | 7.62 | 10.73 | 6.09 | 8.80 | 8.73 | 5.65 | 8.60 | 7.86 |
| B124 | 1.96 | 2.23 | 6.89 | 12.04 | 7.77 | 11.94 | 10.56 | 6.35 | 9.29 | 9.84 |
| B125 | 8.40 | 7.48 | 8.49 | 6.75 | 6.55 | 5.04 | 8.25 | 4.57 | 6.71 | 5.54 |
| B128 | 9.70 | 9.29 | 7.32 | 6.01 | 5.09 | 4.25 | 5.49 | 1.46 | 4.19 | 3.41 |
| Note: p<0.001 for each miRNA, Student’s *t* test | | | | | | | | | | |
