# Supplementary material for: MicroRNA Expression Signatures of Bladder Cancer Revealed by Deep Sequencing
Source: PLoS One. 2011 Mar 28;6(3):e18286. doi: 10.1371/journal.pone.0018286 (PMC3065473; doi:10.1371/journal.pone.0018286)
Supplement: Table S4 — Patient information in deep sequencing set. (DOC) [file pone.0018286.s004.doc]

**Table S4** Clinicopathological information of 9 bladder urothelial carcinoma patients in deep sequencing set

| Patient Number | Sex | Age（years） | Primary or recurrent tumor | Grade | Stage |
| --- | --- | --- | --- | --- | --- |
| B2 | M | 55 | Primary | Low | Ⅰ |
| B4 | M | 42 | recurrent | Low | Ⅱ |
| B5 | M | 53 | Primary | High | Ⅱ |
| B12 | M | 66 | recurrent | Low | Ⅰ |
| B13 | M | 64 | Primary | Low | Ⅱ |
| B15 | M | 40 | recurrent | Low | Ⅱ |
| B 16 | M | 72 | Primary | High | Ⅱ |
| B 20 | M | 66 | Primary | High | Ⅰ |
| B 30 | M | 72 | Primary | High | Ⅳ |
