# Supplementary material for: MicroRNA Expression Signatures of Bladder Cancer Revealed by Deep Sequencing
Source: PLoS One. 2011 Mar 28;6(3):e18286. doi: 10.1371/journal.pone.0018286 (PMC3065473; doi:10.1371/journal.pone.0018286)
Supplement: Table S5 — Primer catalog. (DOC) [file pone.0018286.s005.doc]

**Table S5.** The Catalog Numbers of All-in-One™ miRNA qPCR Primers

| miRNA name | miRNA registation number | Description | Catalog number |
| --- | --- | --- | --- |
| hsa-miR-182 | MIMAT0000259 | qPCR primer against hsa-miR-182 | [HmiRQP0239](http://www.fulengen.com/product/search/detail.php?prt=22&cid=&key=hsa-miR-182) |
| hsa-miR-183 | MIMAT0000261 | qPCR primer against hsa-miR-183 | [HmiRQP0244](http://www.fulengen.com/product/search/detail.php?prt=22&cid=&key=hsa-miR-183) |
| hsa-miR-200a | MIMAT0000682 | qPCR primer against hsa-miR-200a | [HmiRQP0298](http://www.fulengen.com/product/search/detail.php?prt=22&cid=&key=hsa-miR-200a) |
| hsa-miR-143 | MIMAT0000435 | qPCR primer against hsa-miR-143 | [HmiRQP0188](http://www.fulengen.com/product/search/detail.php?prt=22&cid=&key=hsa-miR-143) |
| hsa-miR-195 | MIMAT0000461 | qPCR primer against hsa-miR-195 | [HmiRQP0283](http://www.fulengen.com/product/search/detail.php?prt=22&cid=&key=hsa-miR-195) |
| snRNA U6 |  | qPCR primer against snRNA U6 | HmiRQP9001 |

Note: GeneCopoiea did not provide sequence information of the All-in-One™ miRNA qPCR Primers.
